# Supplementary material for: Differential neutralization and inhibition of SARS-CoV-2 variants by antibodies elicited by COVID-19 mRNA vaccines
Source: Nat Commun. 2022 Jul 27;13:4350. doi: 10.1038/s41467-022-31929-6 (PMC9328008; doi:10.1038/s41467-022-31929-6)
Supplement: Supplementary file 1 — Supplementary Information [file 41467_2022_31929_MOESM1_ESM.pdf]

Supplementary Table 1. List of sequence confirmed mutations present in the spike protein of the viruses used in this study

Variant WHO Label	Signature residue/ Pango Lineage	Mutations in Reporter Virus Spike*	Mutations in Clinical Isolates*
N/A	614D	Reference Spike (SARS-CoV-2/Wuhan-Hu-1/2019)	Reference spike (SARS-CoV-2/USA-WA1/2020)
N/A	614G	D614G	N/A
Alpha	B.1.1.7	H69-, V70-, Y144-, N501Y, A570D, D614G, P681H, T716I, S982A, D1118H	H69-, V70-, Y144-, N501Y, A570D, D614G, P681H, T716I, S982A, D1118H
Beta	B.1.351	L18F, D80A, D215G, L241-, L242-, A243-, R246I, K417N, E484K, N501Y, D614G, A701V	D80A, D215G, L241-, L242-, A243-, K417N, E484K, N501Y, D614G, A701V
Gamma	P.1	L18F, T20N, P26S, D138Y, R190S, K417T, E484K, N501Y, D614G, H655Y, T1027I, V1176F	L18F, T20N, P26S, D138Y, R190S, K417T, E484K, N501Y, D614G, H655Y, T1027I, V1176F
Delta	B.1.617.2	T19R, K77T, G142D, E156-, F157-, R158G, L452R, T478K, D614G, P681R, D950N	T19R, G142D, E156-, F157-, R158G, L452R, T478K, D614G, P681R, D950N
Epsilon	B.1.427/B.1.429	S13I, W152C, L452R, D614G	N/A
Zeta	P.2	E484K, D614G, V1176F	N/A
Eta	B.1.525	Q52R, A67V, H69-, V70-, Y144-, E484K, D614G, Q677H, F888L	N/A
Theta	P.3	L141-, G142-, V143-, A243-, L244-, Y265C, E484K, N501Y, D614G, P681H, E1092K, H1101Y, V1176F	N/A
Iota	B.1.526	L5F, T95I, D253G, E484K, D614G, A701V	N/A
Kappa	B.1.617.1	T95I, G142D, E154K, L452R, E484Q, D614G, P681R, Q1071H	N/A
Lambda	C.37	G75V, T76I, R246-, S247-, Y248-, L249-, T250-, P251-, G252-, D253N, L452Q, F490S, D614G, T859N	G75V, T76I, R246-, S247-, Y248-, L249-, T250-, P251-, G252-, D253N, (T315N), L452Q, F490S, D614G, T859N**
Mu	B.1.621	T95I, Y144S, Y145N, R346K, E484K, N501Y, D614G, P681H, D950N	T95I, Y144S, Y145N, R346K, E484K, N501Y, D614G, P681H, D950N
Omicron	B.1.1.529/BA.1	A67V, H69-, V70-, T95I, G142D, V143-, Y144-, Y145-, N211-,L212I, ins214EPE, G339D, S371L, S373P, S375F, K417N, N440K, G446S, S477N, T478K, E484A, Q493R, G496S, Q498R, N501Y, Y505H, T547K, D614G, H655Y, N679K, P681H, N764K, D796Y, N856K, Q954H, N969K, L981F	A67V, H69-, V70-, T95I, G142D, V143-, Y144-, Y145-, N211-,L212I, ins214EPE, G339D, S371L, S373P, S375F, K417N, N440K, G446S, S477N, T478K, E484A, Q493R, G496S, Q498R, N501Y, Y505H, T547K, D614G, H655Y, N679K, P681H, N764K, D796Y, N856K, Q954H, N969K, L981F
Omicron	B.1.1.529/BA.1.1	N/A	A67V, H69-, V70-, T95I, G142D, V143-, Y144-, Y145-, N211-, L212I, ins214EPE, G339D, R346K, S371L, S373P, S375F, K417N, N440K, G446S, S477N, T478K, E484A, Q493R, G496S, Q498R, N501Y, Y505H, T547K, D614G, H655Y, N679K, P681H, N764K, D796Y, N856K, Q954H, N969K, L981F
Omicron	B.1.1.529/BA.2	T19I, delL24, delP25, delP26, A27S, G142D, V213G, G339D, S371F, S373P, S375F, T376A, D405N, R408S, K417N, N440K, S477N, T478K, E484A, Q493R, Q498R, N501Y, Y505H, D614G, H655Y, N679K, P681H, N764K, D796Y, Q954H, N969K	T19I, delL24, delP25, delP26, A27S, G142D, V213G, G339D, S371F, S373P, S375F, T376A, D405N, R408S, K417N, N440K, S477N, T478K, E484A, Q493R, Q498R, N501Y, Y505H, D614G, H655Y, N679K, P681H, N764K, D796Y, Q954H, N969K

*All viruses were sequenced by Illumina next generation sequencing. Listed are the amino acid mutations detected in the spike gene of the variants, compared to the Wuhan-Hu-1 or USA-WA1 reference spike gene.

** two versions with or without the mutation in parenthesis

N/A, not applicable or not available

Supplementary Table 2. Statistical analysis results – Fig. 2a

Statistical method: two-tailed Wilcoxon matched-pairs signed-rank test

Samples	Number of biologically independent samples	Median of differences	Test statistic (W)	Z value	Effect size (*r*)	Confidence level	Significant?	Summary	P Value
614D vs. 614G	20	-0.01935	14	-0.26133	0.0584	95%	No	ns	8.1236E-01
614D vs. Alpha	20	-131.8	-176	3.2853	0.7346	95%	Yes	***	3.9482E-04
614D vs. Beta	20	-740.7	-210	3.9199	0.8765	95%	Yes	****	1.9073E-06
614D vs. Gamma	20	-529.5	-210	3.9199	0.8765	95%	Yes	****	1.9073E-06
614D vs. Delta	20	-527.3	-210	3.9199	0.8765	95%	Yes	****	1.9073E-06
614D vs. Epsilon	20	-500.9	-210	3.9199	0.8765	95%	Yes	****	1.9073E-06
614D vs. Zeta	20	-623	-210	3.9199	0.8765	95%	Yes	****	1.9073E-06
614D vs. Eta	20	-417.3	-210	3.9199	0.8765	95%	Yes	****	1.9073E-06
614D vs. Theta	20	-709.1	-210	3.9199	0.8765	95%	Yes	****	1.9073E-06
614D vs. Iota	20	-254.8	-204	3.8079	0.8515	95%	Yes	****	9.5367E-06
614D vs. Kappa	20	-649.4	-210	3.9199	0.8765	95%	Yes	****	1.9073E-06
614D vs. Lambda	20	-517.6	-210	3.9199	0.8765	95%	Yes	****	1.9073E-06
614D vs. Mu	20	-730.9	-210	3.9199	0.8765	95%	Yes	****	1.9073E-06
614D vs. Omicron/BA.1	20	-872.5	-210	3.9199	0.8765	95%	Yes	****	1.9073E-06
614D vs. Omicron/BA.2	20	-853.1	-210	3.9199	0.8765	95%	Yes	****	1.9073E-06

ns: not significant

Supplementary Table 2. Statistical analysis results – Fig. 2b

Statistical method: two-tailed Wilcoxon matched-pairs signed-rank test

Samples	Number of biologically independent samples	Median of differences	Test statistic (W)	Z value	Effect size (*r*)	Confidence level	Significant?	Summary	P Value
614D vs. Alpha	26	-29.25	-57	0.72384	0.1420	95%	No	ns	4.8335E-01
614D vs. Beta	26	-451.3	-351	4.4573	0.8741	95%	Yes	****	2.9800E-08
614D vs. Gamma	26	-173.4	-343	4.3558	0.8542	95%	Yes	****	2.0860E-07
614D vs. Delta	26	-240.7	-345	4.3812	0.8592	95%	Yes	****	1.4900E-07
614D vs. Kappa	26	-411	-351	4.4573	0.8741	95%	Yes	****	2.9800E-08
614D vs. Lambda (S1)	26	-42.55	-139	1.7652	0.3462	95%	No	ns	7.9573E-02
614D vs. Lambda (S2)	26	17.4	29	-0.36827	0.0722	95%	No	ns	7.2654E-01
614D vs. Mu	26	-437.1	-351	4.4573	0.8741	95%	Yes	****	2.9800E-08

ns: not significant

Supplementary Table 2. Statistical analysis results – Fig. 2c

Statistical method: two-tailed Wilcoxon matched-pairs signed-rank test

Samples	Number of biologically independent samples	Median of differences	Test statistic (W)	Z value	Effect size (*r*)	Significant?	Summary	P Value
614D vs. Omicron/BA.1	20	-675.5	-210	3.9203	0.8766	Yes	****	1.9073E-06
614D vs. Omicron/BA.1.1	20	-657	-210	3.9199	0.8765	Yes	****	1.9073E-06
614D vs. Omicron/BA.2	20	-672	-210	3.9199	0.8765	Yes	****	1.9073E-06

Supplementary Table 2. Statistical analysis results – Fig. 3a

Statistical method: two-tailed Wilcoxon matched-pairs signed-rank test

Samples	Number of biologically independent samples	Median of differences	Test statistic (W)	Z value	Effect size (*r*)	Significant?	Summary	P Value
614D vs. Beta	20	-49.22	-190	3.5466	0.7930	Yes	***	8.2016E-05
614D vs. Omicron/BA.1	20	-68.37	-210	3.9199	0.8765	Yes	****	1.9073E-06
614D vs. Omicron/BA.2	20	-70.54	-206	3.8453	0.8598	Yes	****	5.7220E-06

Supplementary Table 2. Statistical analysis results – Fig. 3b

Statistical method: two-tailed Wilcoxon matched-pairs signed-rank test

Samples	Number of biologically independent samples	Median of differences	Test statistic (W)	Z value	Effect size (*r*)	Significant?	Summary	P Value
614D vs. Beta	20	-1962	-208	3.8826	0.8682	Yes	****	3.8147E-06
614D vs. Omicron/BA.1	20	-2789	-210	3.9199	0.8765	Yes	****	1.9073E-06
614D vs. Omicron/BA.2	20	-2864	-210	3.9199	0.8765	Yes	****	1.9073E-06

Supplementary Table 2. Statistical analysis results – Fig. 3c

Statistical method: two-tailed Wilcoxon matched-pairs signed-rank test

Samples	Number of biologically independent samples	Median of differences	Test statistic (W)	Z value	Effect size (*r*)	Significant?	Summary	P Value
614D vs. Omicron/BA.1	20	-2837	-210	3.9199	0.8765	Yes	****	1.9073E-06
614D vs. Omicron/BA.1.1	20	-3050	-210	3.9199	0.8765	Yes	****	1.9073E-06
614D vs. Omicron/BA.2	20	-2856	-208	3.8826	0.8682	Yes	****	3.8147E-06

Supplementary Table 2. Statistical analysis results – Fig. 4a

Statistical method: two-tailed Wilcoxon matched-pairs signed-rank test

Samples (pre-booster vs. post booster)	Number of biologically independent samples	Median of differences	Test statistic (W)	Z value	Effect size (*r*)	Significant?	Summary	P Value
N	18	0.3791	75	-1.6331	0.3849	No	ns	0.1084
RBD	20	7995	-210	-3.9199	0.8765	Yes	****	1.9073E-06
S	20	6188	-210	-3.9199	0.8765	Yes	****	1.9073E-06

ns: not significant

Supplementary Table 2. Statistical analysis results – Fig. 4b

Statistical method: two-tailed Wilcoxon matched-pairs signed-rank test

Samples (pre-booster vs. post booster)	Number of biologically independent samples	Median of differences	Test statistic (W)	Z value	Effect size (*r*)	Confidence level	Significant?	Summary	P Value
614D	20	492262	210	-3.9199	0.8765	95%	Yes	****	1.9073E-06
Alpha	20	400899	210	-3.9199	0.8765	95%	Yes	****	1.9073E-06
Beta	20	283298	210	-3.9199	0.8765	95%	Yes	****	1.9073E-06
Omicron/BA.1	17	105630	153	-3.6214	0.8783	95%	Yes	****	1.5259E-05
Omicron/BA.1.1	17	65954	153	-3.6214	0.8783	95%	Yes	****	1.5259E-05
Omicron/BA.2	20	136185	210	-3.9199	0.8765	95%	Yes	****	1.9073E-06

Supplementary Table 2. Statistical analysis results – Fig. 5a

Statistical method: two-tailed Wilcoxon matched-pairs signed-rank test

Samples	Number of biologically independent samples	Median of differences	Test statistic (W)	Z value	Effect size (*r*)	Significant?	Summary	P Value
614D								
Non sera vs. 2x	12	-945200	-78	3.0594	0.8832	Yes	***	4.8828E-04
Non sera vs. 5x	12	-1125769	-78	3.0594	0.8832	Yes	***	4.8828E-04
614G								
Non sera vs. 2x	12	-1987854	-78	3.0594	0.8832	Yes	***	4.8828E-04
Non sera vs. 5x	12	-2566538	-78	3.0594	0.8832	Yes	***	4.8828E-04
Alpha								
Non sera vs. 2x	6	-3413157	-21	2.2014	0.8987	Yes	*	3.1250E-02
Non sera vs. 5x	6	-4172772	-21	2.2014	0.8987	Yes	*	3.1250E-02
Beta								
Non sera vs. 2x	12	-1641944	-64	2.8655	0.8272	Yes	**	1.9531E-03
Non sera vs. 5x	12	-2318020	-70	2.7456	0.7926	Yes	**	3.4180E-03
Gamma								
Non sera vs. 2x	6	-3547374	-21	2.2014	0.8987	Yes	*	3.1250E-02
Non sera vs. 5x	6	-4427088	-21	2.2014	0.8987	Yes	*	3.1250E-02
Delta								
Non sera vs. 2x	12	-779895	-78	3.0594	0.8832	Yes	***	4.8828E-04
Non sera vs. 5x	12	-1405411	-78	3.0594	0.8832	Yes	***	4.8828E-04
Epsilon								
Non sera vs. 2x	6	-400000	-17	1.7821	0.7275	No	ns	9.3750E-02
Non sera vs. 5x	6	-1275000	-21	2.2014	0.8987	Yes	*	3.1250E-02
Zeta								
Non sera vs. 2x	6	-2100000	-21	2.2014	0.8987	Yes	*	3.1250E-02
Non sera vs. 5x	6	-3743000	-21	2.2014	0.8987	Yes	*	3.1250E-02
Eta								
Non sera vs. 2x	6	-562150	-17	1.7821	0.7275	No	ns	9.3750E-02
Non sera vs. 5x	6	-489900	-21	2.2014	0.8987	Yes	*	3.1250E-02
Theta								
Non sera vs. 2x	6	-94454	-21	2.2014	0.8987	Yes	*	3.1250E-02
Non sera vs. 5x	6	-192274	-21	2.2014	0.8987	Yes	*	3.1250E-02
Iota								
Non sera vs. 2x	6	-2103000	-19	1.9917	0.8131	No	ns	6.2500E-02
Non sera vs. 5x	6	-2677500	-21	2.2014	0.8987	Yes	*	3.1250E-02
Kappa								
Non sera vs. 2x	6	-190000	-11	1.1531	0.4708	No	ns	3.1250E-01

Non sera vs. 5x	6	-1120000	-21	2.2014	0.8987	Yes	*	3.1250E-02
Lambda								
Non sera vs. 2x	6	-2745000	-21	2.2014	0.8987	Yes	*	3.1250E-02
Non sera vs. 5x	6	-2180000	-21	2.2014	0.8987	Yes	*	3.1250E-02
Mu								
Non sera vs. 2x	6	-624113	-15	1.4832	0.6055	No	ns	1.5625E-01
Non sera vs. 5x	6	-1249484	-21	2.0226	0.8257	Yes	*	3.1250E-02
Omicron/BA.1								
Non sera vs. 2x	6	257580	8	-0.84094	0.3433	No	ns	4.6875E-01
Non sera vs. 5x	6	286200	15	-1.5724	0.6419	No	ns	1.5625E-01

ns: not significant

Supplementary Table 2. Statistical analysis results – Fig. 5b

Statistical method: two-tailed Wilcoxon matched-pairs signed-rank test

Samples	Number of biologically independent samples	Median of differences	Test statistic (W)	Z value	Effect size (*r*)	Significant?	Summary	P Value
Pre-booster sera								
614D								
No sera vs. 2 dose, 10X	18	-492200	-171	3.724	0.877755218	Yes	****	7.6294E-06
No sera vs. 2 dose, 20X	18	-492800	-171	3.724	0.877755218	Yes	****	7.6294E-06
Omicron/BA.1								
No sera vs. 2 dose, 10X	18	-326800	-171	3.724	0.877755218	Yes	****	7.6294E-06
No sera vs. 2 dose, 20X	18	-387950	-171	3.724	0.877755218	Yes	****	7.6294E-06
Omicron/BA.2								
No sera vs. 2 dose, 10X	18	-235015	-171	3.724	0.877755218	Yes	****	7.6294E-06
No sera vs. 2 dose, 20X	18	-251230	-171	3.724	0.877755218	Yes	****	7.6294E-06

.

Supplementary Table 2. Statistical analysis results – Fig. 5c

Statistical method: two-tailed Wilcoxon matched-pairs signed-rank test

Samples	Number of biologically independent samples	Median of differences	Test statistic (W)	Z value	Effect size (*r*)	Significant?	Summary	P Value
Post-booster sera								
614D								
No sera vs. 2 dose, 10X	18	-305140	-171	3.724	0.877755218	Yes	****	7.6294E-06
No sera vs. 2 dose, 20X	18	-305260	-171	3.724	0.877755218	Yes	****	7.6294E-06
Omicron/BA.1								
No sera vs. 2 dose, 10X	18	-282040	-171	3.724	0.877755218	Yes	****	7.6294E-06
No sera vs. 2 dose, 20X	18	-286440	-171	3.724	0.877755218	Yes	****	7.6294E-06
Omicron/BA.2								
No sera vs. 2 dose, 10X	18	-221640	-171	3.724	0.877755218	Yes	****	7.6294E-06
No sera vs. 2 dose, 20X	18	-225560	-171	3.724	0.877755218	Yes	****	7.6294E-06
