# Supplementary material for: Differential neutralization and inhibition of SARS-CoV-2 variants by antibodies elicited by COVID-19 mRNA vaccines
Source: Nat Commun. 2022 Jul 27;13:4350. doi: 10.1038/s41467-022-31929-6 (PMC9328008; doi:10.1038/s41467-022-31929-6)
Supplement: Supplementary file 3 — Reporting Summary [file 41467_2022_31929_MOESM3_ESM.pdf]

nature portfolio

Corresponding author(s): Bin Zhou
David Wentworth

Last updated by author(s): Jun 23, 2022

Reporting Summary

Nature Portfolio wishes to improve the reproducibility of the work that we publish. This form provides structure for consistency and transparency in reporting. For further information on Nature Portfolio policies, see our Editorial Policies and the Editorial Policy Checklist.

Statistics

For all statistical analyses, confirm that the following items are present in the figure legend, table legend, main text, or Methods section.

n/a	Confirmed	
☐	☒	The exact sample size (*n*) for each experimental group/condition, given as a discrete number and unit of measurement
☐	☒	A statement on whether measurements were taken from distinct samples or whether the same sample was measured repeatedly
☐	☒	The statistical test(s) used AND whether they are one- or two-sided<br>*Only common tests should be described solely by name; describe more complex techniques in the Methods section.*
☒	☐	A description of all covariates tested
☐	☒	A description of any assumptions or corrections, such as tests of normality and adjustment for multiple comparisons
☐	☒	A full description of the statistical parameters including central tendency (e.g. means) or other basic estimates (e.g. regression coefficient) AND variation (e.g. standard deviation) or associated estimates of uncertainty (e.g. confidence intervals)
☐	☒	For null hypothesis testing, the test statistic (e.g. *F*, *t*, *r*) with confidence intervals, effect sizes, degrees of freedom and *P* value noted<br>*Give P values as exact values whenever suitable.*
☒	☐	For Bayesian analysis, information on the choice of priors and Markov chain Monte Carlo settings
☒	☐	For hierarchical and complex designs, identification of the appropriate level for tests and full reporting of outcomes
☐	☒	Estimates of effect sizes (e.g. Cohen's *d*, Pearson's *r*), indicating how they were calculated

Our web collection on statistics for biologists contains articles on many of the points above.

Software and code

Policy information about availability of computer code

Data collection	Plates were scanned using a CellInsight CX5 High-Content Screening Platform (Thermo Scientific) running an 'Acquisition Only' protocol within Cellomics Scan Version 6.6.0 (Thermo Scientific, Build 8153). Foci were identified and quantified using appropriate 'Spot Detection' protocol within Cellomics Scan Version 6.6.2 (Thermo Scientific, Build 8533) for reporter virus-based assay or a BioTek Cytation3 instrument and Gen5 software for clinical isolate-based assay.
Data analysis	Spot counts for each channel were exported for further analysis in R (Version 4.0.3). The R script has been deposited in GitHub: https://github.com/CDCgov/SARS-CoV-2_FRNTcalculations/ as described in Code Availability. Graphs were made by GraphPad Prism (Version 8.4.2). Statistical analysis were performed using GraphPad Prism 9.3.1 and R version 4.1.2, with P value < 0.05 considered significant. GraphPad Prism v7.04 was used for determining the FRNT50 value of clinical isolate-based assay.

For manuscripts utilizing custom algorithms or software that are central to the research but not yet described in published literature, software must be made available to editors and reviewers. We strongly encourage code deposition in a community repository (e.g. GitHub). See the Nature Portfolio guidelines for submitting code & software for further information.

Data

Policy information about availability of data

All manuscripts must include a data availability statement. This statement should provide the following information, where applicable:

- Accession codes, unique identifiers, or web links for publicly available datasets
- A description of any restrictions on data availability
- For clinical datasets or third party data, please ensure that the statement adheres to our policy

The full genome sequences of viruses generated in this study have been deposited in the GenBank database under accession code ON571504-ON571519 (https://

www.ncbi.nlm.nih.gov/nuccore/ON571504-ON571519) as indicated in the Data Availability. Data supporting the findings of this study have been provided in the Source data file.

Field-specific reporting

Please select the one below that is the best fit for your research. If you are not sure, read the appropriate sections before making your selection.

- [x] Life sciences
- [ ] Behavioural & social sciences
- [ ] Ecological, evolutionary & environmental sciences

For a reference copy of the document with all sections, see nature.com/documents/nr-reporting-summary-flat.pdf

Life sciences study design

All studies must disclose on these points even when the disclosure is negative.

Sample size	No sample size calculations were performed. Twenty to twenty-six post-second dose sera (2-6 weeks post second dose), 20 pre-booster sera (6-7 months post-second dose) and 20-25 post-booster sera (2-6 weeks post-third dose) were used based on the availability which meet the selection criteria (vaccine history), and not based on power calculations. 20 sera or even fewer number of sera are commonly used in SARS-CoV-2 neutralization assays.
Data exclusions	No data was excluded.
Replication	Each serum sample was diluted in sextuplicate for each run of neutralization assay. 20 or more different sera were used in each run and the titers from 1-8 runs were used to calculate the geometric mean FRNT50 titers. For virus replication assay, 2 different pooled sera (each from 10 individual serum samples) were used in 3-9 technical replications with 1 or 2 independent runs. All attempts of replication were successful. MSD immunoassay was performed in duplicates with different dilutions and the averaged results were reported in the study.
Randomization	Randomization was not applied since serum samples were selected based on vaccine history, and viruses were selected and used based on national or international authorities' designation (VOC/VOI). The procedures in this study such as virus isolation, neutralization assay do not require randomization.
Blinding	Patient information was blinded in the study. All the serum samples/viruses were assigned with CDC specific name codes (deidentified). The investigators were blinded sample identity during data collection and/or analysis.

Reporting for specific materials, systems and methods

We require information from authors about some types of materials, experimental systems and methods used in many studies. Here, indicate whether each material, system or method listed is relevant to your study. If you are not sure if a list item applies to your research, read the appropriate section before selecting a response.

Materials & experimental systems

n/a	Involved in the study	
☐	☒	Antibodies
☐	☒	Eukaryotic cell lines
☒	☐	Palaeontology and archaeology
☒	☐	Animals and other organisms
☐	☒	Human research participants
☒	☐	Clinical data
☒	☐	Dual use research of concern

Methods

n/a	Involved in the study	
☒	☐	ChIP-seq
☒	☐	Flow cytometry
☒	☐	MRI-based neuroimaging

Antibodies

Antibodies used	SARS/SARS-CoV-2 Nucleocapsid Monoclonal Antibody (Invitrogen, Cat No: MA5-29981) used as primary antibody (1:4,000 dilution) in clinical isolate-based assay; Goat anti-Mouse IgG (H+L) Highly Cross-Adsorbed Secondary Antibody, Alexa Fluor™ Plus 647 (Invitrogen, Cat No: A32728) used as secondary antibody (1:400 dilution) in clinical isolate-based neutralization assay.
Validation	All antibodies used were obtained from commercial vendors and specificity characteristics were based on descriptions and information provided by the manufacturers.<br>Links below to manufacturer pages with statements where given and/or original papers where antibodies used are first described.<br>https://www.thermofisher.com/antibody/product/SARS-SARS-CoV-2-Nucleocapsid-Antibody-clone-5-Monoclonal/MA5-29981<br>https://www.thermofisher.com/antibody/product/Goat-anti-Mouse-IgG-H-L-Highly-Cross-Adsorbed-Secondary-Antibody-Polyclonal/A32728

Eukaryotic cell lines

Policy information about cell lines

Cell line source(s)	Vero E6 cells (ATCC, CRL-1586); VeroE6/TMPRSS2 cells25 (JCRB Cell Bank, JCRB1819); Calu-3 cells (ATCC, HTB-55)
Authentication	None. All cells were used within 10 passages after they were obtained.
Mycoplasma contamination	All cell lines were tested negative for mycoplasma.
Commonly misidentified lines (See ICLAC register)	No misidentified cell lines were used in the study

Human research participants

Policy information about studies involving human research participants

Population characteristics	Participants were US adults (18-65 years old, including both male and female) having no prior or current diagnosis of infection with SARS-CoV-2 and were fully vaccinated (at least 14 days after the second dose or the third dose) with either Pfizer-BioNTech mRNA vaccine BNT162b2 or Moderna mRNA-1273 vaccine.
Recruitment	Vaccinee serum samples were collected from individuals through the Influenza and Other Viruses in the Acutely Ill (IVY) Network, a Centers for Disease Control and Prevention (CDC)-funded collaboration to monitor the effectiveness of SARS-CoV-2 vaccines among US adults. The individuals should not have prior or current diagnosis of infection with SARS-CoV-2 and not have any COVID-19 like symptoms at the time of sample collection.
Ethics oversight	This activity was approved by each participating institution, either as a research project with written informed consent or as a public health surveillance project without written informed consent. This activity was also reviewed by the CDC and conducted in a manner consistent with applicable federal laws and CDC policies.

Note that full information on the approval of the study protocol must also be provided in the manuscript.
